# The importance of ultrasound in identifying and differentiating patients with early inflammatory arthritis: a narrative review

**DOI:** 10.1186/s13075-019-2050-4

**Published:** 2020-01-02

**Authors:** Gurjit S. Kaeley, Catherine Bakewell, Atul Deodhar

**Affiliations:** 1 Division of Rheumatology and Clinical Immunology, University of Florida College of Medicine, 653-1 West 8th St., LRC 2nd Floor L-14, Jacksonville, FL 32209 USA; 2 Intermountain Healthcare, Salt Lake City, UT USA; 3 Division of Arthritis and Rheumatic Diseases, Oregon Health & Science University, Portland, OR USA

**Keywords:** Ultrasound, Inflammatory arthritis, Synovitis, Enthesitis, Bone erosions, Imaging

## Abstract

Early differentiation between different types of inflammatory arthritis and subsequent initiation of modern treatments can improve patient outcomes by reducing disease activity and preventing joint damage. Routine clinical evaluation, laboratory testing, and radiographs are typically sufficient for differentiating between inflammatory and predominantly degenerative arthritis (e.g., osteoarthritis). However, in some patients with inflammatory arthritis, these techniques fail to accurately identify the type of early-stage disease. Further evaluation by ultrasound imaging can delineate the inflammatory arthritis phenotype present. Ultrasound is a noninvasive, cost-effective method that enables the evaluation of several joints at the same time, including functional assessments. Further, ultrasound can visualize pathophysiological changes such as synovitis, tenosynovitis, enthesitis, bone erosions, and crystal deposits at a subclinical level, which makes it an effective technique to identify and differentiate most common types of inflammatory arthritis. Limitations associated with ultrasound imaging should be considered for its use in the differentiation and diagnosis of inflammatory arthritides.

## Introduction

The development and progression of inflammatory arthritis depends on both environmental and genetic factors and can affect an estimated 115 to 271 people per 100,000 adults [1, 2]. Symptoms of joint, tendon, or entheseal inflammation can be either short lived or persistent. If inflammation continues, permanent skeletal damage can occur, leading to morbidity and disability [3]. The advent of the modern treatment armamentarium and treat-to-target strategies now makes rapid evaluation and accurate diagnosis in patients with inflammatory arthritis important. Specifically, early treatment with targeted therapies can alter long-term outcomes by minimizing disease activity, preventing joint damage and disability, and improving patients’ quality of life [2, 4–6].

Routine clinical evaluations that consist of a thorough history and physical examination, laboratory testing, and plain radiography can often establish the presence of arthritis. However, it can sometimes be challenging to differentiate between inflammatory and degenerative causes of arthritis, especially when clinical signs are sparse and serologies are negative. Initially, it is important to determine if a patient has inflammatory arthritis or a predominantly degenerative arthritis such as osteoarthritis. Subsequently, the patient should be evaluated to determine the type of inflammatory arthritis if inflammation is the suspected cause of joint pain. Common inflammatory joint disorders in adults include crystal-induced arthritis, rheumatoid arthritis (RA), and spondyloarthritis (SpA) including psoriatic arthritis (PsA), reactive arthritis, enteropathic arthritis, and ankylosing spondylitis (AS) [2]. Additionally, inflammatory arthritis or bursitis in older patients (≥ 50 years of age) may be a result of polymyalgia rheumatica (PMR) or crystalline arthropathies [7–9]. Common symptoms of inflammatory arthritis may include joint swelling, erythema, morning stiffness longer than 0.5–1 h, and radiographic evidence of bone loss around joints [10, 11]. The number of joints involved, the type of joints involved (e.g., small vs large), and the pattern of joint involvement (e.g., symmetrical vs asymmetrical) can also be similar between arthritides [1, 12–14]. Further, unique disease manifestations, such as enthesitis and dactylitis in obese patients, can be clinically challenging to detect [15]. Additionally, serologies may fail to conclusively differentiate between these diseases and elevation of acute-phase reactants is nonspecific [16–19].

**Fig. 1:**
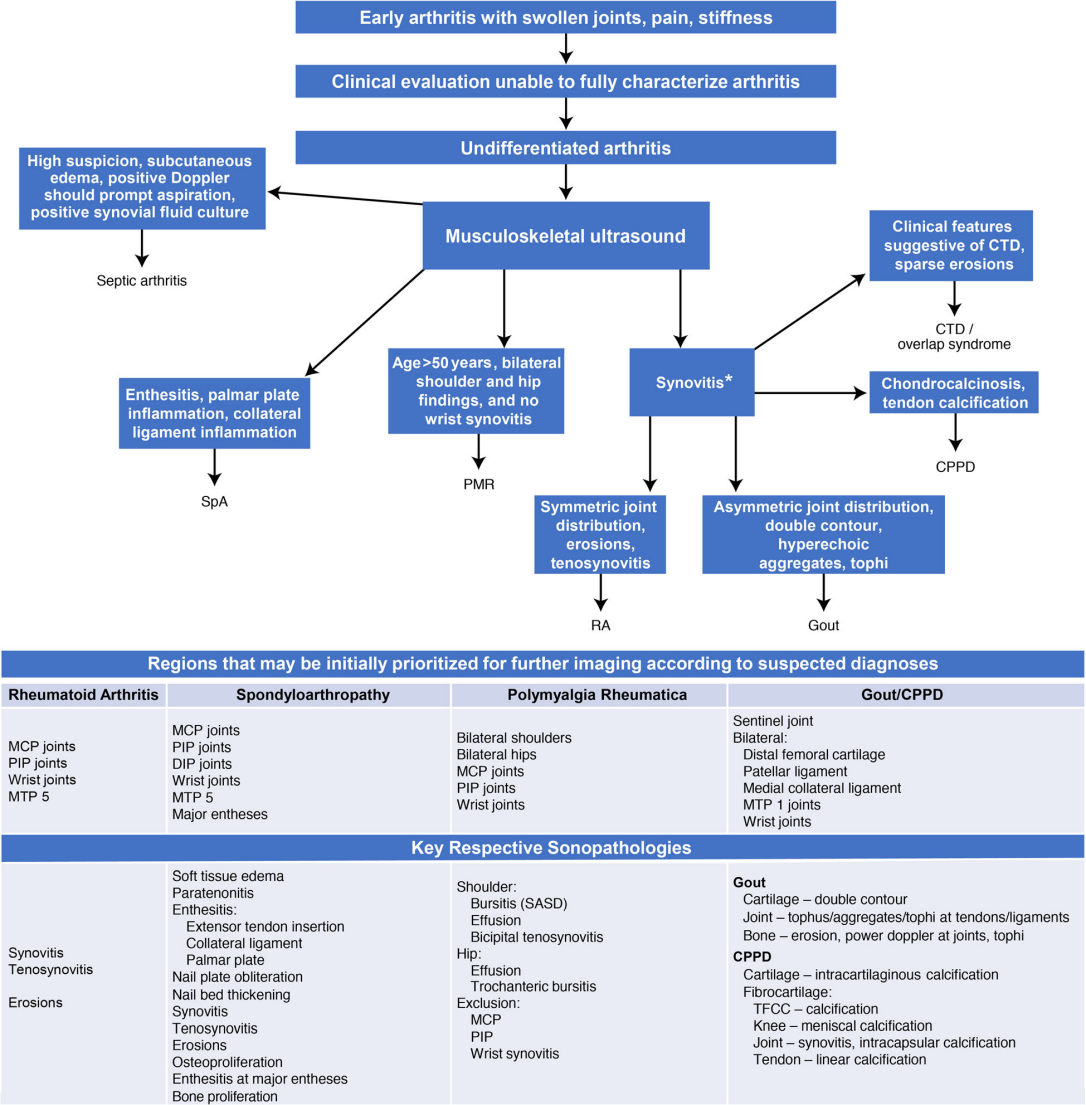
Use of ultrasound in diagnostic decision making. This algorithm was developed by the authors and was not based on a clinical study. Note: *Osteoarthritis can cause synovitis but is excluded from this algorithm. CPPD, calcium pyrophosphate deposition; CTD, connective tissue disorder; MCP, metacarpophalangeal; MTP, metatarsophalangeal, PIP, proximal interphalangeal; PMR, polymyalgia rheumatica; RA, rheumatoid arthritis; SASD, subacromial-subdeltoid; SpA, spondyloarthritis; TFCC, triangular fibrocartilage complex

During early disease and in patients with milder symptoms, in whom clinical findings are not definitive, imaging is needed to accurately differentiate between different types of inflammatory arthritis. European League Against Rheumatism (EULAR) recommendations for the management of early arthritis are guided by an overarching principle that “a definitive diagnosis in a patient with early arthritis should only be made after a careful history taking and clinical examination, which should also guide laboratory testing and additional procedures” [20]. In our opinion, imaging, just like clinical examination, needs to be considered in the context of clinical presentation, with possible differential diagnoses taking demographic characteristics into account (Fig. 1). In current practice, ultrasound, magnetic resonance imaging (MRI) techniques, and, to a lesser extent, computed tomography (CT) are generally regarded as superior to conventional radiographs for identifying and differentiating early signs of inflammatory arthritis [21]. While MRI and CT are useful, CT is limited by exposure to ionizing radiation and MRI is limited by high cost and limited availability. Ultrasound, a nonionizing imaging technique, is often preferred because many musculoskeletal structures can be examined, it can be performed at the point-of-care, and it can be used on patients for whom MRI is contraindicated. In patients with inflammatory arthritis, ultrasound can detect important clues such as subclinical synovitis, asymptomatic entheseal inflammation, bone erosions, and crystal deposits, which could otherwise be missed in physical examinations [4, 22–28]. The importance of ultrasound has also been highlighted by its inclusion in the two most recent EULAR/American College of Rheumatology (ACR) classification criteria for PMR and gout [29, 30].

Ultrasound can be used to generate high-resolution images of joints, tendons, entheses, synovia, cartilage, bursae, bony cortex, nails, and soft-tissue vascularity. Structures can be imaged in a dynamic, multiplanar fashion, allowing for visualization of synovial changes, joint effusions, tendon tears, and bone erosions [24, 31, 32]. Musculoskeletal ultrasound can be used to differentiate trauma-related injuries that can initially mimic arthritis, including muscle lesions, occult fractures, and tendon rupture or subluxation [28]. Power Doppler ultrasound (PDUS) imaging is used to assess soft tissue and nerve lesions, tissue vascularization, and hyperemia of synovial structures, tendons, and entheses [4, 33, 34]. The inability of ultrasound to penetrate bone surface and, hence, visualize bone structures is an important limitation to bear in mind, which may be addressed using correlative plain X-rays. Additionally, only a small number of studies have examined how ultrasound should be integrated to the process of diagnosis for inflammatory arthritis.

This manuscript provides an in-depth review of how ultrasound—a portable, convenient, noninvasive, and cost-effective imaging technique—can be used in the differential diagnosis of early inflammatory arthritis phenotypes (Table 1) and also assesses any important limitations of the technique. The authors also propose an algorithm (Fig. 1) that may enable working through a differential diagnosis both clinically and by prioritizing anatomical targets.

**Table 1:** Ultrasound features used in differentiation of early inflammatory arthritis

Rheumatoid arthritis	Spondyloarthritis	Crystal arthropathies	Polymyalgia rheumatica	Septic arthritis
• Joint effusion, synovial proliferation, synovial pannus, and hyperemia in typical RA distribution • Tenosynovial effusions, synovial hypertrophy, and hyperemia • Cortical bone erosions and cartilage lesions • Multijoint assessments confirming typical distribution of involvement	• Enthesitis characterized by tendon/ligament hypoechogenicity and thickening, calcification, bone erosions, intralesional focal calcification or fibrous tissue, and abnormal vascularization at enthesis insertion on power Doppler ultrasound • Cortical bone erosions and enthesophytes (heterogeneous to RA) • Synovitis and tenosynovitis • Confounding factors: age, BMI	• Tophaceous deposits: • Cartilage: double contour sign (gout) • Periarticular: heterogeneous collection in soft tissue, “snowstorm” appearance sometimes with anechoic rim • Tendons and ligaments: intratendinous tophi and ovoid-shaped microdeposits with hyperechoic densities • Cortical bone erosions • CPPD deposits: • Hyaline cartilage: hyperechoic, within the layer of cartilage • Fibrocartilage: hyperechoic, rounded or amorphous deposits • Basic calcium phosphate: • Hyperechoic foci with variable acoustic shadowing • Hyperemia on Doppler	• Bilateral subacromial/subdeltoid bursitis • Biceps long-head tenosynovitis • Trochanteric bursitis • Synovitis • Hip effusion • Less common findings include enthesitis, glenohumeral effusions, flexor tenosynovitis, and peripheral synovitis • Should not have hand- or wrist-joint synovitis	• Joint effusion, sometimes with hyperechogenicity and heterogeneity • Increased peri-synovial vascularity with color Doppler • Ultrasound can guide joint aspiration • Clinical suspicion has the highest priority

*BMI* body mass index, *CPPD* calcium pyrophosphate dehydrate, *RA* rheumatoid arthritis

### Ultrasonographic evaluation in suspected inflammatory arthritis

#### Synovitis and tenosynovitis

**Fig. 2:**
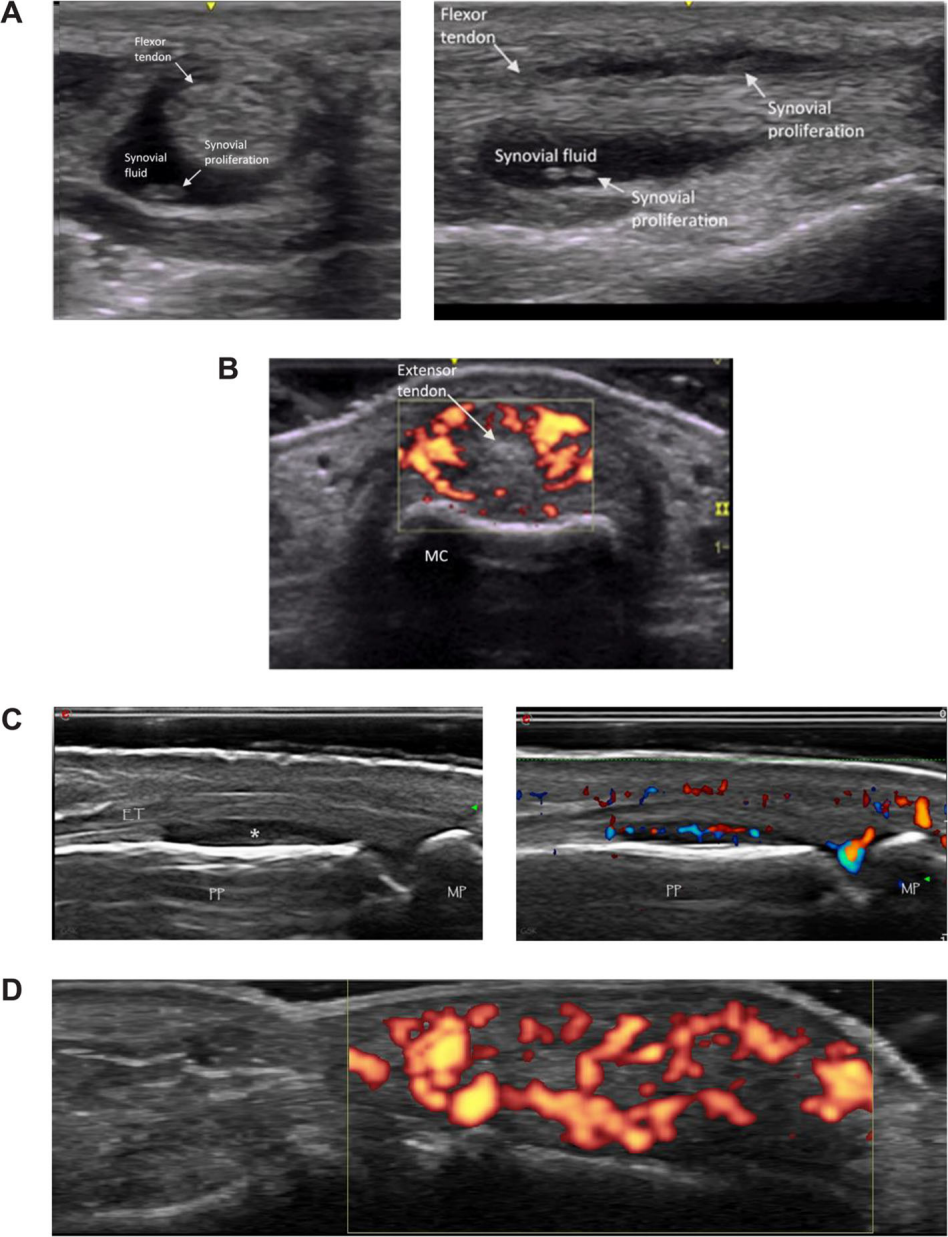
Ultrasound imaging of synovitis and tenosynovitis. **a** Flexor tenosynovitis in transverse (left) and longitudinal (right) views. **b** Metacarpophalangeal joint paratenonitis, dorsal aspect of second metacarpophalangeal joint. MC, metacarpal. **c** Dorsal proximal interphalangeal B-mode (left) and power Doppler (right) images indicating synovitis in the recess (asterisk). PP, proximal phalanx; MP, middle phalanx; ET, extensor digitorum tendon. **d** Positive power Doppler signal of finger pulp

Among the key features in diagnosing inflammatory arthritis is the presence of synovitis as well as the distribution of joints involved. In mild or early-onset inflammatory arthritis, it may be difficult to discern clinical synovitis. Similarly, mild tenosynovitis may not be clinically apparent. Synovitis and tenosynovitis are common features of early RA and SpA (Fig. 2a–d). Synovitis is characterized on grayscale ultrasound by intra-articular tissue that is abnormally thickened, hypoechoic or anechoic (relative to subdermal fat), nondisplaceable, and poorly compressible [26]. As synovial proliferation progresses, articular cartilage becomes disrupted, and erosions can be observed at the osteochondral junction [4].

Tenosynovitis is characterized by hypoechoic or anechoic thickened tissue with or without fluid in the tendon sheath [26] and is not a specific lesion. Presence of synovial hypertrophy should prompt the use of PDUS or color Doppler to establish vascularity and, hence, inflammation of the tissue. The degree of Doppler sensitivity of the user’s equipment should be known to avoid false negative testing. Doppler imaging findings need to be taken into context with the overall clinical picture, and the operator should recognize the pitfalls of false positive and false negative results. Doppler sensitivity can be gauged by the degree of vascularity of the distal finger pulp (Fig. 2d), with Doppler signal in more than one third of the finger indicating a reasonable sensitivity of the machine and settings. Thus, sonographic signs of synovitis should include both synovial hypertrophy and vascularity.

The value of ultrasound in identifying subclinical synovitis has been demonstrated by finding synovitis in asymptomatic joints of patients with early oligoarthritis that led to the reclassification of oligoarthritis as polyarthritis for many patients [4, 22, 23]. In patients with arthralgia not diagnosed with inflammatory arthritis, the absence of ultrasound-detected synovitis is associated with a high (89%) negative predictive value for the development of inflammatory arthritis over 1 year [35].

**Fig. 3:**
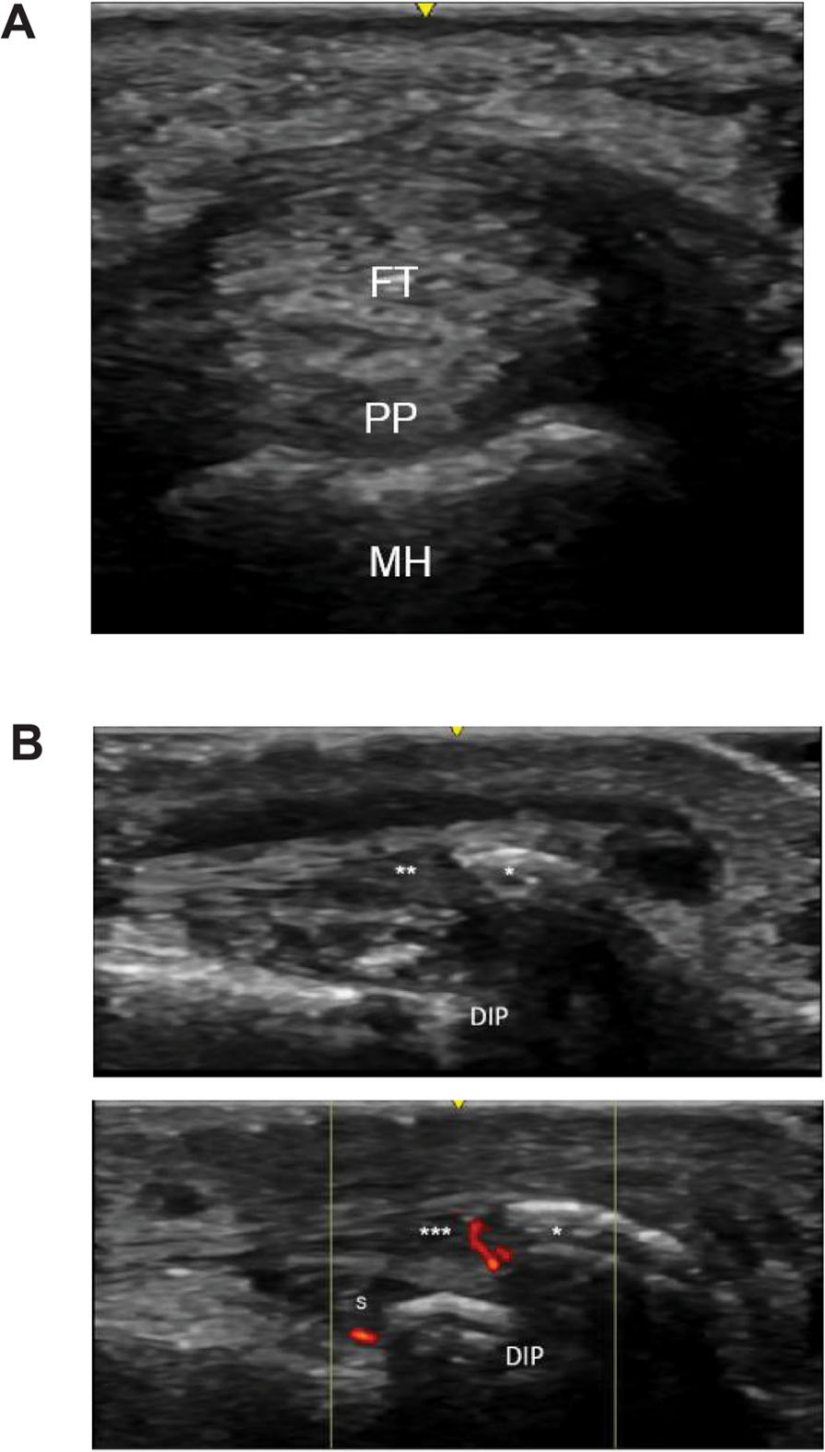
Ultrasound findings for differentiation of psoriatic arthritis from rheumatoid arthritis. **a** Short-axis view of palmar plate inflammation. FT, flexor tendon; MH, metacarpal head; PP, palmar plate. **b** Dorsal long view of enthesitis of the extensor tendon from a distal interphalangeal joint in a patient with psoriatic arthritis. DIP, distal interphalangeal; S, DIP synovitis; asterisk (*), enthesophyte; double asterisks (**), extensor tendon demonstrating thickening, hypoechogenicity, and loss of fibrillar architecture; triple asterisks (***), extensor tendon with insertional Doppler

Features of RA that can be visualized on ultrasound include rheumatoid nodules and synovial cysts, as well as common secondary complications, such as median nerve entrapment in the carpal tunnel [36]. Additionally, the distribution of joint involvement may help differentiate RA from PsA as, for example, synovitis of the distal interphalangeal joints is characteristic of PsA rather than RA [33]. Synovial hypertrophy in the finger joints of patients with RA can be particularly well characterized with ultrasound by comprehensively examining palmar and dorsal aspects of proximal interphalangeal and metacarpophalangeal joints. In RA, synovial hypertrophy is most often detected at the dorsal metacarpophalangeal joints and palmar aspect of the proximal interphalangeal joints [37]. However, if the diagnosis is in question, then both dorsal and palmar aspects should be examined to evaluate signs of tendonitis and palmar plate enthesitis. MRI studies of patients with dactylitis have shown increased signal at the palmar plate and there is some discussion that this may be a form of enthesitis [38]. In a study of patients with early PsA and RA, Zabotti et al. [39] found that synovitis was observed more frequently in patients with RA. In patients with early PsA, periarticular soft-tissue edema, metacarpophalangeal joint peri-extensor tenonitis, and proximal interphalangeal joint extensor tendon enthesitis were found more often [39]. Palmar plate inflammation (Fig. 3a), digital enthesitis (Fig. 3b), and collateral ligament enthesitis may also help differentiate PsA from RA. Diffuse extensor paratenonitis and flexor tenosynovitis (Fig. 2b) is also observed in patients with PsA dactylitis.

These features can serve as additional differentiating factors between early SpA and RA [24, 39]. Paratenonitis (defined as the lack of a sheath on the extensor tendon above the metacarpophalangeal joint with accompanying inflammatory changes to the extensor tendon consisting of increased thickness, loss of fibrillar architecture, and increased power Doppler signal) may also be found in established RA [39, 40]. Flexor tenosynovitis is strongly associated with dactylitis, which occurs in 32 to 48% of patients with PsA [41], and along with joint synovitis, flexor tenosynovitis is among the most commonly reported features of dactylitic digits. Other reported dactylitic tissue changes visible by ultrasound include soft-tissue thickening or edema, osteoproliferation, and sesamoid abnormalities [42]. Although these sonographic features have been well documented in patients with clinically obvious dactylitis, their presence in a patient with early inflammatory arthritis may help differentiate early PsA from RA.

Imaging findings need to be correlated with clinical presentation and suspected differential diagnoses. For example, synovitis can be the result of lupus, gout, or osteoarthritis [27, 43–45], but imaging findings can narrow the differential diagnoses considerably. An important limitation is the awareness of findings in apparently normal populations. Recently, studies have demonstrated the prevalence of ultrasound-detected joint inflammatory abnormalities (synovial effusion and/or synovial hypertrophy) in the hands and feet of healthy individuals. In a study of 207 healthy individuals, 6621 joints were analyzed and 9% had at least 1 ultrasound abnormality [46]. However, B-mode findings with PDUS score of > 2 only occurred in a minority of patients. Further, because this was a cross-sectional study, it is not clear if these individuals had onset of early inflammatory arthritis. Thus, care needs to be exercised in interpreting imaging findings in patients with minimal symptoms and should be considered in the overall clinical context.

#### Enthesitis

Enthesitis is a hallmark clinical feature of SpA, especially PsA, and is observed less frequently in other inflammatory arthritides, such as RA. Entheseal inflammation is often asymptomatic and may be overlooked on clinical examination [24]. For example, Balint and colleagues [47] found that in a study of 35 patients with SpA, clinical examination identified enthesitis in 22% (75/348) of sites compared with 56% (195/348) of sites on ultrasound examination. Ultrasound examination has also been used to demonstrate that nail disease in PsA and psoriasis is associated with distal interphalangeal enthesopathy [48].

**Fig. 4:**
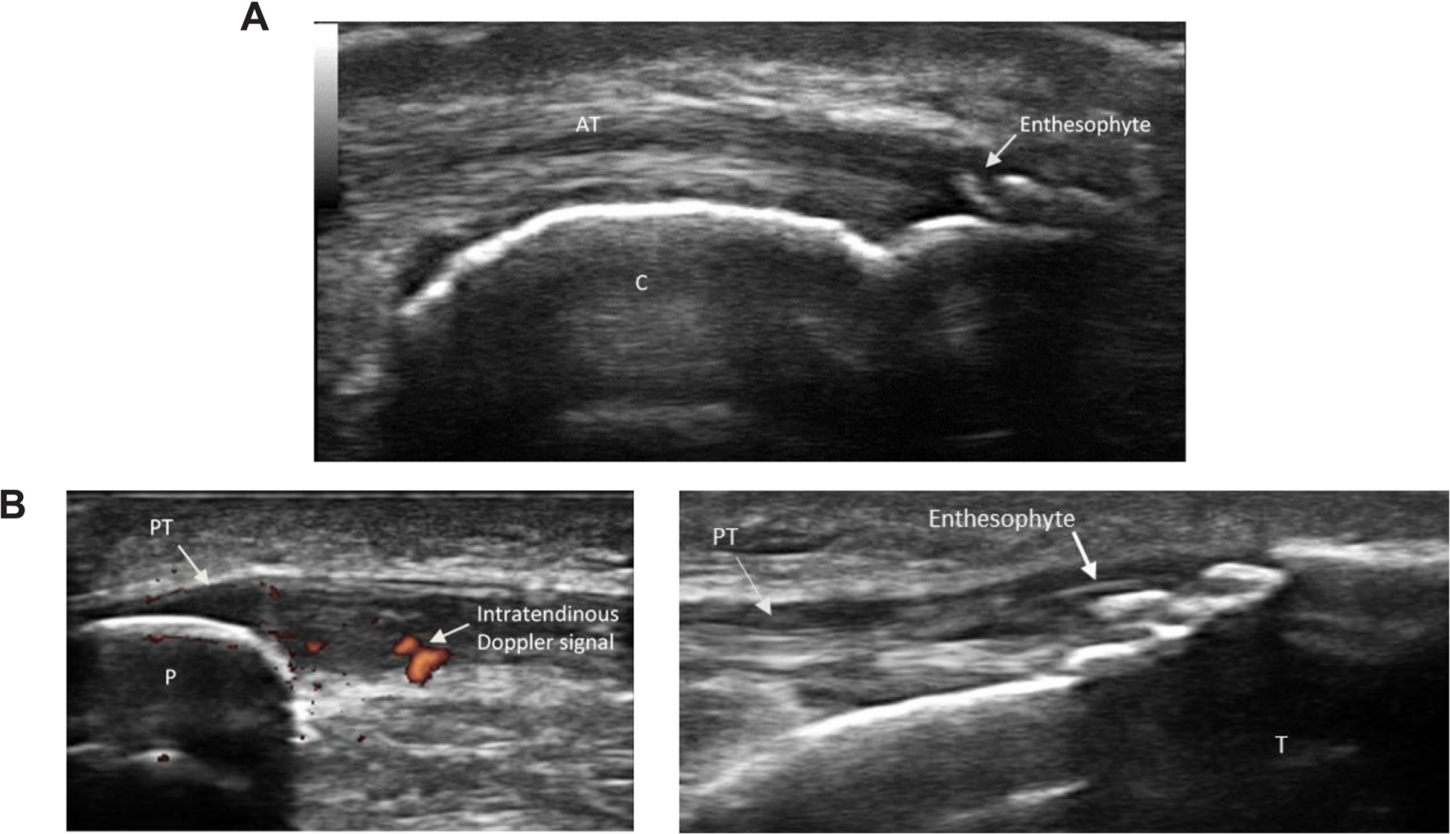
Ultrasound imaging of enthesitis. **a** Achilles enthesophyte in a patient with spondyloarthritis. AT, Achilles tendon; C, calcaneus. **b** Patellar enthesitis in a patient with psoriatic arthritis. Left, Doppler with abnormal intratendinous signal; right, enthesophyte. P, patella; PT, patellar tendon/ligament; T, tibia

Sonography can depict not only echotexture changes (such as loss of fibrillar echotexture and tendon thickening) at the enthesis but also peri-entheseal Doppler signal. It can also demonstrate pathologic changes in the adjacent tissues, such as proximal tendinopathy, bone erosions, and bursitis. In many cases, ultrasound can be used to visualize subclinical enthesitis that cannot be detected with physical examination [49]. Inactive or chronic enthesitis may manifest as tendon thickening, bulky enthesophytes, intratendinous calcification, and bone erosions [26, 32]. Further, in PsA, the severity of sonographic enthesitis is associated with peripheral and axial joint damage [50]. Entheseal ultrasound assessment should include longitudinal and transverse scans with tendons in both neutral and flexed positions [25]. A flexed position may provide better visualization of grayscale abnormalities, but may create tension that diminishes a Doppler signal [51]. Although there is some controversy about which entheses should be evaluated by ultrasound when a diagnosis of SpA is suspected, inclusion of the Achilles tendon and selection of the knee (quadriceps and patellar) and plantar fascia entheses are typically recommended (Fig. 4) [52]. Assessment of Achilles entheses, however, should be approached with caution as age, body mass index, and regular physical exercise have all been associated with structural damage on ultrasound in PsA [53].

A hallmark of inflammatory peripheral enthesitis seen with PDUS is vascularization at cortical bone insertion. The Outcome Measures in Rheumatology (OMERACT) ultrasound subgroup proposes the inclusion of enthesitis as part of an outcome measure only when the visualized signal is within 2 mm of the bony cortex [54]. Other groups, such as Group for Research and Assessment of Psoriasis and Psoriatic Arthritis (GRAPPA) ultrasound committee, have proposed scoring separately in proximal and distal entheses as well as bursa and to test the relative specificity of site-specific Doppler signals in subjects with and without enthesitis [55]. This will enable a data-driven approach to establishing the most sensitive and specific combination of findings associated with a diagnosis of spondyloarthritis at the patient level. Regardless, detection of any vascularized entheses by PDUS is a sensitive and specific characteristic for diagnosis of SpA [56]. Nearby structures should also be evaluated because adjacent bursitis and tendon calcification are commonly observed by ultrasound at sites of enthesitis [52, 57].

While not used in routine clinical practice, ultrasound enthesitis scoring systems have been studied as tools for diagnostic classification of SpA [57, 58]. The most common scoring systems are the Glasgow Ultrasound Enthesitis Scoring System (GUESS) and the MAdrid Sonographic Enthesitis Index (MASEI) [15, 25]. GUESS combines grayscale ultrasound evaluations of 5 lower-limb entheseal sites, while MASEI combines grayscale and PDUS evaluations of 6 upper- and lower-extremity entheseal sites [25]. In a cross-sectional study that evaluated 25 patients with SpA and 29 matched controls, de Miguel and colleagues [58] found that a MASEI score of ≥ 18 could be used with specificity of 82.8% as a cutoff to differentiate between cases of SpA and healthy controls. In a separate study of 113 patients with early SpA and 57 matched controls, de Miguel and colleagues found that a MASEI cutoff score of ≥ 20 had specificity of 89.5% [59]. However, age and body mass index are significantly correlated with GUESS and MASEI scores, and degenerative or mechanical abnormalities in weight-bearing joints may be incorrectly identified as inflammatory arthritis, especially in obese patients for whom excess weight puts added mechanical stress on lower limb entheses [15, 60, 61]. Recent literature has focused on examination of hand entheses to differentiate between early RA and PsA. Zabotti et al. [39] found that extensor tendon tenonitis, extensor slip enthesitis, and periarticular edema were useful in differentiating PsA from RA. However, extensor slip abnormalities can also be seen in patients with osteoarthritis [62] and RA [40].

#### Bone erosions

**Fig. 5:**
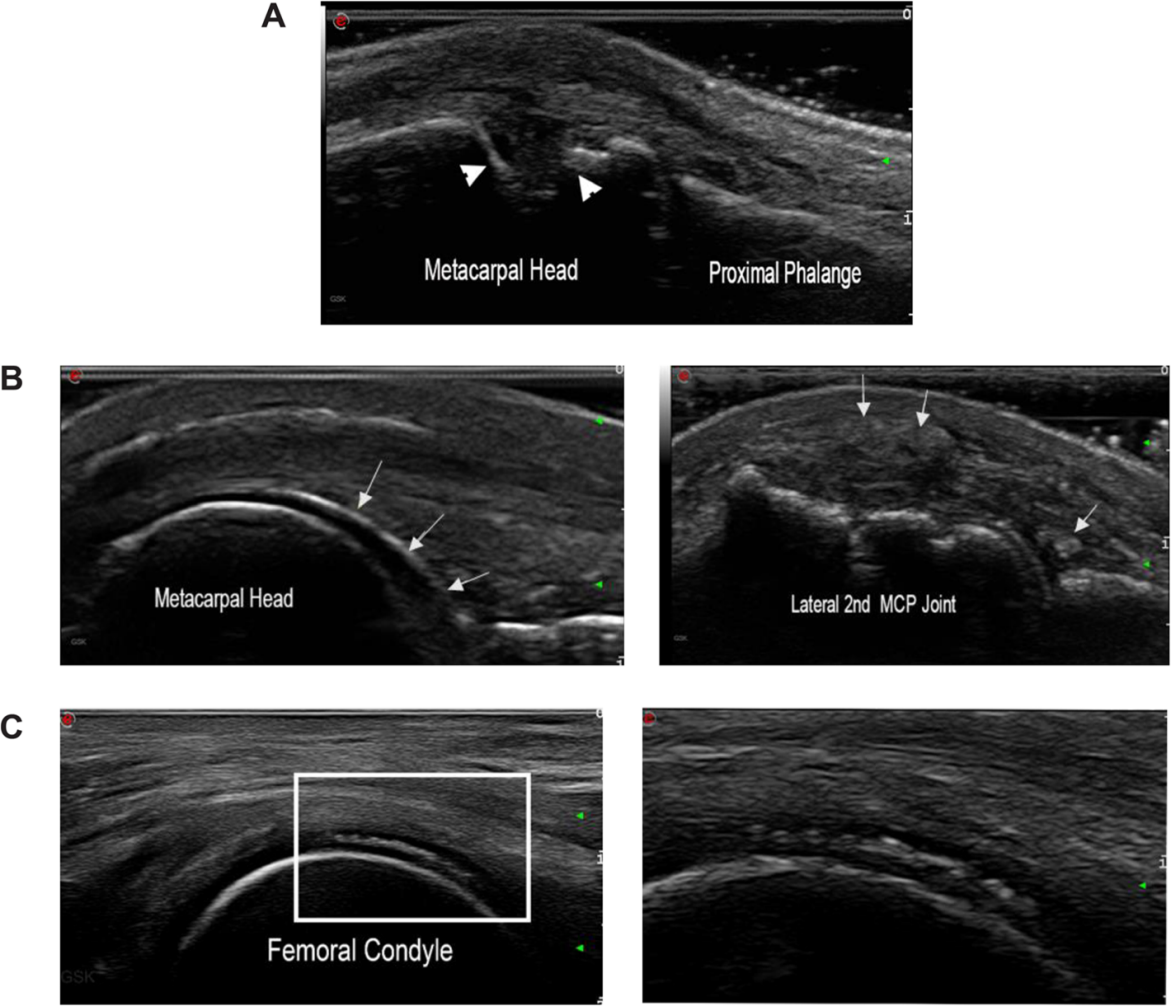
Ultrasound imaging of bone erosions and crystal deposits. **a** Transverse view of second metacarpophalangeal joint in a patient with rheumatoid arthritis; arrowheads denote bone erosion. **b** Left, chondrosynovial urate deposition at the second metacarpophalangeal joint (arrows); right, at the same joint, intra- and peri-articular tophaceous deposits seen as heterogeneous collections (arrows). **c** Left, calcium pyrophosphate crystal deposition seen sandwiched within the cartilage; right, magnified view of the white rectangular area on the left

Bone erosion is an important hallmark of both RA and SpA that can be identified with ultrasound (Fig. 5) based on intra-articular discontinuity of the bone surface that is visible in two perpendicular planes [4, 26]. Ultrasound can be used to accurately identify cortical irregularities of at least 2 mm in width as breaks in the bone surface associated with inflammatory arthritis [63]. Ultrasonic detection of bone erosions is more feasible in hand and toe joints than in bones with poor ultrasound windows, such as carpal and tarsal bones [4]. In a recent study, joint erosions were predominantly found in patients with RA (91.4%), followed by gout (90.0%), PsA (75.0%), osteoarthritis (70.0%), and finally healthy volunteers (33.3%) [64]. Although the mere presence of ultrasound-detected erosions may not be specific for RA, larger erosions at the second and fifth metacarpophalangeal joints, fifth metatarsophalangeal joint, and distal ulna may sway the diagnosis towards RA [64]. Further, in patients with RA whose PDUS synovitis activity and clinical disease activity are well controlled, the detection of bone erosion with ultrasound after discontinuation of biologic disease-modifying antirheumatic drugs may be a risk factor for relapse [65].

Distinguishing between bone erosions from physiologic cortical breaks that are not caused by inflammation is important. These false-positive ultrasound findings are typically a result of small lesions (< 2 mm) or lesions found at the palmar grooves of the metacarpal heads and phalangeal bases, where nutrient blood vessels pass through vascular bone channels and enter the bone marrow. Another source of false-positive findings are pseudo-erosions formed by osteophytes in forceps-like arrangements, which are common in patients with PsA and can make it difficult to visualize the cortical bone surface [63].

#### Crystal deposits

Ultrasound can uniquely demonstrate the differential chondrosynovial deposition of urate in comparison to intra-cartilaginous chondrocalcinosis (Fig. 5) [4, 27, 28].

Tophi within soft tissues and tendons can be identified as heterogeneous collections with hyperechoic dots, and frequently with anechoic rims [4, 66]. These may be clinically undetectable and yet cause significant symptoms when involved in a flareup. Careful examination of the symptomatic areas may help in detecting these deposits and thus assist in diagnosing a patient with episodic arthralgia. In calcium pyrophosphate dehydrate crystal deposition disease, tendon calcifications can also be observed as well as classical chondrocalcinosis in multiple joints [4, 67]. OMERACT ultrasound definitions of calcium pyrophosphate dehydrate crystal deposition disease provided reliable results in the hyaline cartilage and fibrocartilage of the knee—the most frequently involved site in the disease—however, the definitions were not as reliable at other anatomical sites [68]. In a subsequent study which evaluated a wider range of anatomical locations, OMERACT ultrasound definitions of calcium pyrophosphate dehydrate crystal deposition disease were reliable for the triangular fibrocartilage of the wrist and the acromioclavicular joint [69]. As with other imaging modalities, the presence of chondrocalcinosis does not imply calcium pyrophosphate dehydrate crystal deposition disease and careful clinical correlation needs to occur. Ultrasound plays an active role in many procedures, including guided needle placement for the location and safe aspiration of synovial fluid to obtain a definitive crystal analysis [70]. Further, in addition to helping diagnose crystal deposits, ultrasound is sensitive to changes in gout and can be used to monitor tophus burden [71].

#### Considerations in older patients

In older patients presenting with shoulder and hip pain, a diagnosis of PMR should be considered. Ultrasound features that are suggestive of a differential diagnosis of PMR include bilateral subacromial and subdeltoid bursitis, long biceps tendon tenosynovitis, trochanteric bursitis, and glenohumeral and hip effusion [7, 28, 72].

In developing the 2012 ACR/EULAR classification criteria for PMR, evaluation of scoring criteria in 125 patients with new-onset PMR and 169 controls showed that adding ultrasound measures to the scoring system increased specificity for discriminating PMR from other mimicking conditions such as elderly-onset RA (EORA) from 78 to 81% [29]. A subsequent systematic literature review by Sakellariou and colleagues [73] found that bilateral shoulder bursitis on ultrasound had the highest specificity of any individual finding for diagnosis of PMR. The absence of synovial proliferation at the hand or wrist on ultrasound is also suggestive of PMR rather than EORA [28]. Negative serologic testing for rheumatoid factor or anticitrullinated protein antibodies can also help rule out a diagnosis of EORA [7].

## Conclusions

With increasing availability of biologic therapies that target specific disease pathogenesis, it is more important than ever for clinicians to be able to differentiate between different types of inflammatory arthritis. Subsequent differentiation of the specific phenotype of inflammatory arthritis present can be complicated by ambiguity in the clinical picture and laboratory findings not allowing for a clear diagnosis. Consequently, imaging—especially ultrasound—is now an essential part of early inflammatory arthritis diagnosis and differentiation, and its inclusion in two ACR/EULAR classification criteria highlights its importance [29, 30] despite the limited number of studies that have examined how ultrasound should be integrated to the diagnostic process for inflammatory arthritis. Furthermore, detection of subclinical deposits of tophi is often an epiphany in patients with episodic seronegative arthralgia. Thus, ultrasound has become a valuable tool in the hands of an experienced clinician in evaluating patients with arthralgia who have sparse clinical signs.

## Data Availability

Data sharing is not applicable to this article as no datasets were generated or analyzed during the current study.

## Abbreviations

ACR: American College of Rheumatology
AS: Ankylosing spondylitis
CT: Computed tomography
EORA: Elderly-onset RA
EULAR: European League Against Rheumatism
GRAPPA: Group for Research and Assessment of Psoriasis and Psoriatic Arthritis
GUESS: Glasgow Ultrasound Enthesitis Scoring System
MASEI: MAdrid Sonographic Enthesitis Index
MRI: Magnetic resonance imaging
OMERACT: Outcome Measures in Rheumatology
PDUS: Power Doppler ultrasound
PMR: Polymyalgia rheumatica
PsA: Psoriatic arthritis
RA: Rheumatoid arthritis
SpA: Spondyloarthritis
